# Short-term Dynamics after Single- and Three-piece Acrylic Intraocular Lens Implantation: A Swept-source Anterior Segment Optical Coherence Tomography Study

**DOI:** 10.1038/s41598-018-28609-1

**Published:** 2018-07-06

**Authors:** Tatsuhiko Sato, Shunsuke Shibata, Motoaki Yoshida, Ken Hayashi

**Affiliations:** Hayashi Eye Hospital, Fukuoka, Japan

## Abstract

Accurate alignment of an intraocular lens (IOL) is indispensable for achieving accurate postoperative refractive outcomes. Thus, we evaluated decentration and tilt of single- and three-piece IOLs, as well as anterior chamber depth (ACD), at 3 hours, 24 hours, 2 weeks, and 4 weeks after cataract surgery, using swept-source anterior segment optical coherence tomography. There was no significant difference in postoperative visual acuity between eyes with single- or three-piece IOLs. Absolute values of IOL decentration at 24 hours and 2 weeks after surgery were significantly larger (*P* = 0.008 and 0.046, respectively) in eyes with the single-piece IOL than in those with the three-piece IOL. Both single- and three-piece IOLs tended to tilt toward the inferotemporal direction; however, there was no significant difference in the absolute values of IOL tilt at any postoperative time point. ACD at 24 hours after surgery was significantly deeper (*P* = 0.009) in eyes with the three-piece IOL, compared with eyes with the single-piece IOL. Therefore, although both single- and three-piece IOL locations varied transiently after surgery, IOL locations were similar between both IOLs at 4 weeks after surgery and were not associated with any statistical difference in visual function.

## Introduction

Accurate alignment of an intraocular lens (IOL) after cataract surgery is indispensable for achieving an accurate postoperative refractive power. In a previous study, we compared IOL stabilities using Scheimpflug videophotography and found that the degrees of IOL decentration and tilt in eyes with a single-piece IOL were similar to those observed in eyes with a three-piece IOL^1^. We also showed a significant shallowing of the anterior chamber depth (ACD) after three-piece IOL implantation, resulting in an approximately 0.4-diopter (D) myopic shift in spherical equivalent (SE)^1^.

A second generation of swept-source anterior segment optical coherence tomography (SS-ASOCT) has become commercially available since 2015 (CASIA2, Tomey Corp., Nagoya, Japan); this new instrument possesses 13-mm depth and 16-mm width of scan range, along with ≤ 10 µm of axial resolution when utilizing a light whose wavelength is 1310 nm. Recent studies have reported that ASOCT serves as a useful tool in evaluation of IOL location and stability, as well as in measurement of ACD^2,3^. In addition, ASOCT demonstrates high repeatability for measurement of IOL stability, compared with conventional methods (e.g., Purkinje images and the Scheimpflug method)^4,5^.

In this study, we used SS-ASOCT to investigate short-term dynamics after single- and three-piece IOL implantation procedures, utilizing identical IOL material from a single manufacturer, after uneventful phacoemulsification.

## Results

### Patients

A total of 58 patients were screened for inclusion. Three patients declined to participate in this study and five patients did not meet the inclusion criteria. Ultimately, 50 patients were enrolled in this study. Mean age of the patients was 68.9 ± 5.3 years. There were 29 women (58%) and 21 men (42%). Mean axial length was 24.37 ± 1.80 mm (range: 21.81–29.26 mm).

### Intraobserver and Interobserver Reproducibility

Intraobserver reproducibility is shown in Table 1. In eyes with the single-piece IOL, intraclass correlation coefficients (ICCs) were 0.972–0.979 for the degree of IOL decentration, 0.965–0.974 for the degree of IOL tilt, and 0.999 for the ACD. In eyes with the three-piece IOL, the ICCs were 0.982–0.985 for the degree of IOL decentration, 0.823–0.898 for the degree of IOL tilt, and 0.999 for the ACD.

**Table 1:** Intraobserver Correlation Coefficient in Eyes with Single- and Three-piece IOL.

Single-piece		Decentration		Tilt		ACD	
Examiner		ICC	95% Confidence Interval	ICC	95% Confidence Interval	ICC	95% Confidence Interval
1	ICC (1,1)	0.975	0.950–0.989	0.972	0.945–0.988	0.999	0.997–0.999
2	ICC (1,1)	0.972	0.946–0.988	0.965	0.931–0.985	0.999	0.998–1.000
3	ICC (1,1)	0.979	0.959–0.991	0.974	0.949–0.989	0.999	0.999–1.000
**Three-piece**		**Decentration**		**Tilt**		**ACD**	
**Examiner**		**ICC**	**95% Confidence Interval**	**ICC**	**95% Confidence Interval**	**ICC**	**95% Confidence Interval**
1	ICC (1,1)	0.985	0.970–0.994	0.856	0.741–0.936	0.999	0.998–1.000
2	ICC (1,1)	0.984	0.968–0.993	0.898	0.812–0.956	0.999	0.998–1.000
3	ICC (1,1)	0.982	0.965–0.993	0.823	0.690–0.921	0.999	0.998–1.000

Interobserver reproducibility is shown in Table 2. ICCs in eyes with the single-piece IOL were 0.988 for the degree of IOL decentration, 0.960 for the degree of IOL tilt, and 0.998 for the ACD, whereas those in eyes with the three-piece IOL were 0.982 for the degree of IOL decentration, 0.858 for the degree of IOL tilt, and 0.998 for the ACD.

**Table 2:** Interobserver Correlation Coefficient in Eyes with Single- and Three-piece IOL.

Single-piece	Decentration		Tilt		ACD	
	ICC	95% Confidence Interval	ICC	95% Confidence Interval	ICC	95% Confidence Interval
ICC(2,1)	0.988	0.973–0.995	0.960	0.915–0.984	0.998	0.995–0.999
**Three-piece**	**Decentration**		**Tilt**		**ACD**	
	**ICC**	**95% Confidence Interval**	**ICC**	**95% Confidence Interval**	**ICC**	**95% Confidence Interval**
ICC(2,1)	0.982	0.962–0.993	0.858	0.716–0.941	0.998	0.995–0.999

According to a previous study^6^, all ICC values in this study were judged as almost perfect.

### Intraocular Lens Decentration

**Figure 1:**
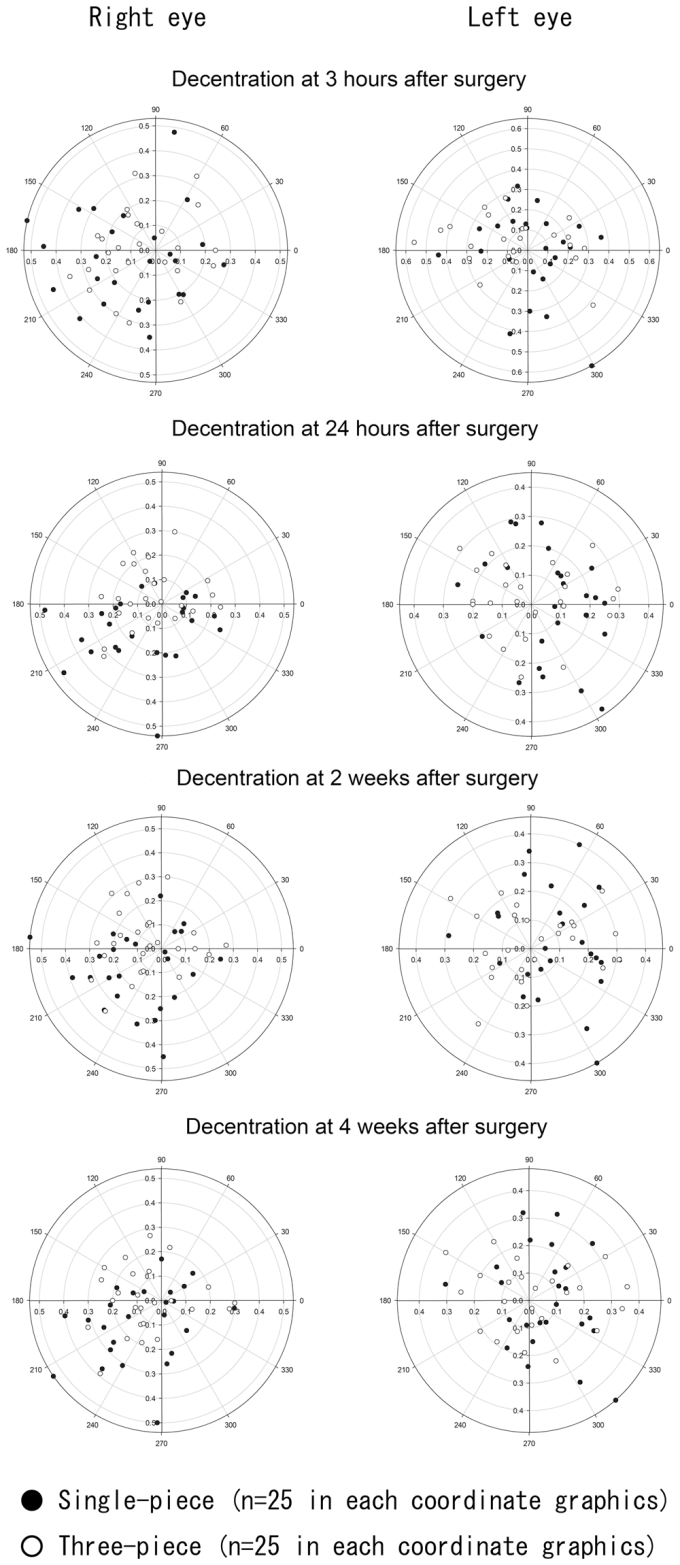
Decentration of single- and three-piece intraocular lenses (IOLs) at 3 hours, 24 hours, 2 weeks, and 4 weeks after surgery. Coordinate graphics on the right and left sides show IOL decentration in the left and right eyes, respectively.

Figure 1 shows the distribution of IOL decentration in eyes with both single- and three-piece IOLs, using the method described by Holladay *et al*.^7^ In both eyes with single- and three-piece IOLs, there were no significant differences in postoperative absolute values and azimuths of IOL decentration. Absolute values of IOL decentration at 24 hours and 2 weeks after surgery were significantly larger (*P* = 0.008 and 0.046, respectively) in eyes with a single-piece IOL than in eyes with a three-piece IOL (Table 3).

**Table 3:** Postoperative Changes of IOL Decentration.

	Single-piece IOL (n = 50)	Three-piece IOL (n = 50)	*P* value
Absolute Value (mm, average ± SD)			
3 hours	0.25 ± 0.13	0.22 ± 0.11	0.210
24 hours	0.22 ± 0.11	0.17 ± 0.08	0.008
2 weeks	0.23 ± 0.11	0.18 ± 0.09	0.046
4 weeks	0.21 ± 0.12	0.19 ± 0.09	0.170
Azimuth (degree, average ± SD)			
3 hours	180.5 ± 102.9	161.1 ± 90.7	0.330
24 hours	191.8 ± 113.7	168.2 ± 100.8	0.350
2 weeks	195.0 ± 107.3	151.8 ± 97.1	0.052
4 weeks	201.4 ± 105.5	183.9 ± 100.4	0.465

IOL: intraocular lens, SD: standard deviation.

### Intraocular Lens Tilt

**Figure 2:**
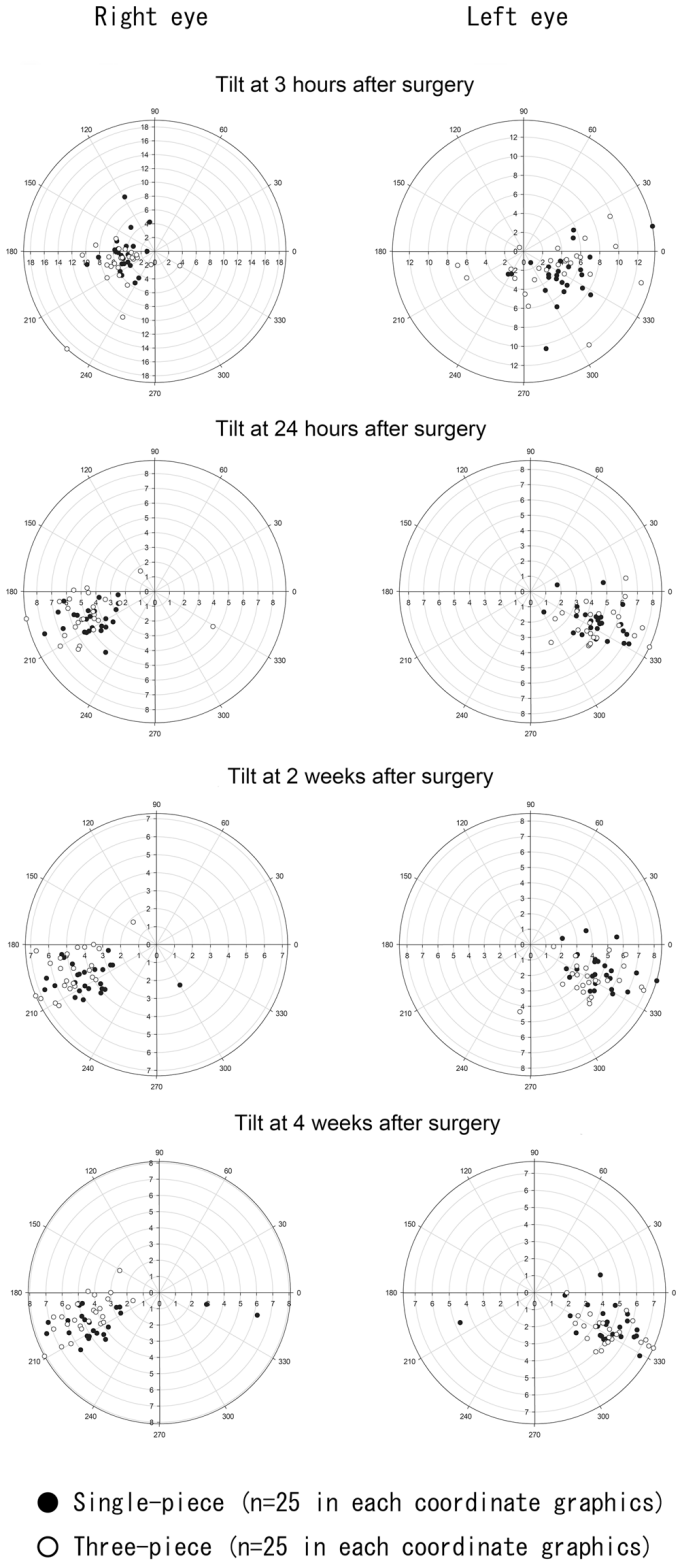
Tilt of single- and three-piece intraocular lenses (IOLs) at 3 hours, 24 hours, 2 weeks, and 4 weeks after surgery. Coordinate graphics on the right and left sides show IOL tilt in the left and right eyes, respectively.

Figure 2 shows the distribution of IOL tilt in eyes with both single- and three-piece IOLs, using the method described by Holladay *et al*.^7^ Both IOLs tilted toward the inferotemporal direction, relative to the visual axis. In both eyes with single- and three-piece IOLs, there were no significant differences in postoperative absolute values of IOL tilt. In contrast, there was a significant difference (*P* = <0.001) in the postoperative azimuth of single-piece IOL tilt, and the azimuths of single-piece IOLs at 24 hours, 2 weeks, and 4 weeks after surgery were significantly (*P* < 0.05) larger than azimuths at 3 hours after surgery. The azimuth of IOL tilt at 4 weeks postoperatively was significantly larger (*P* = <0.001) in eyes with the single-piece IOL than in eyes with the three-piece IOL (Table 4).

**Table 4:** Postoperative Changes of IOL Tilt.

	Single-piece IOL (n = 50)	Three-piece IOL (n = 50)	*P* value
Absolute Value (degree, average ± SD)			
3 hours	5.39 ± 2.21	5.81 ± 3.25	0.510
24 hours	4.84 ± 1.37	5.10 ± 1.51	0.320
2 weeks	4.65 ± 1.30	4.85 ± 1.37	0.440
4 weeks	4.80 ± 1.24	4.91 ± 1.45	0.650
Azimuth (degree, average ± SD)			
3 hours	231.7 ± 89.3	227.9 ± 91.3	0.670
24 hours	255.4 ± 81.7	259.1 ± 78.7	0.230
2 weeks	252.6 ± 89.1	262.1 ± 70.0	0.051
4 weeks	267.0 ± 75.7	256.5 ± 78.1	<0.001

IOL: intraocular lens, SD: standard deviation.

### Anterior Chamber Depth

**Figure 3:**
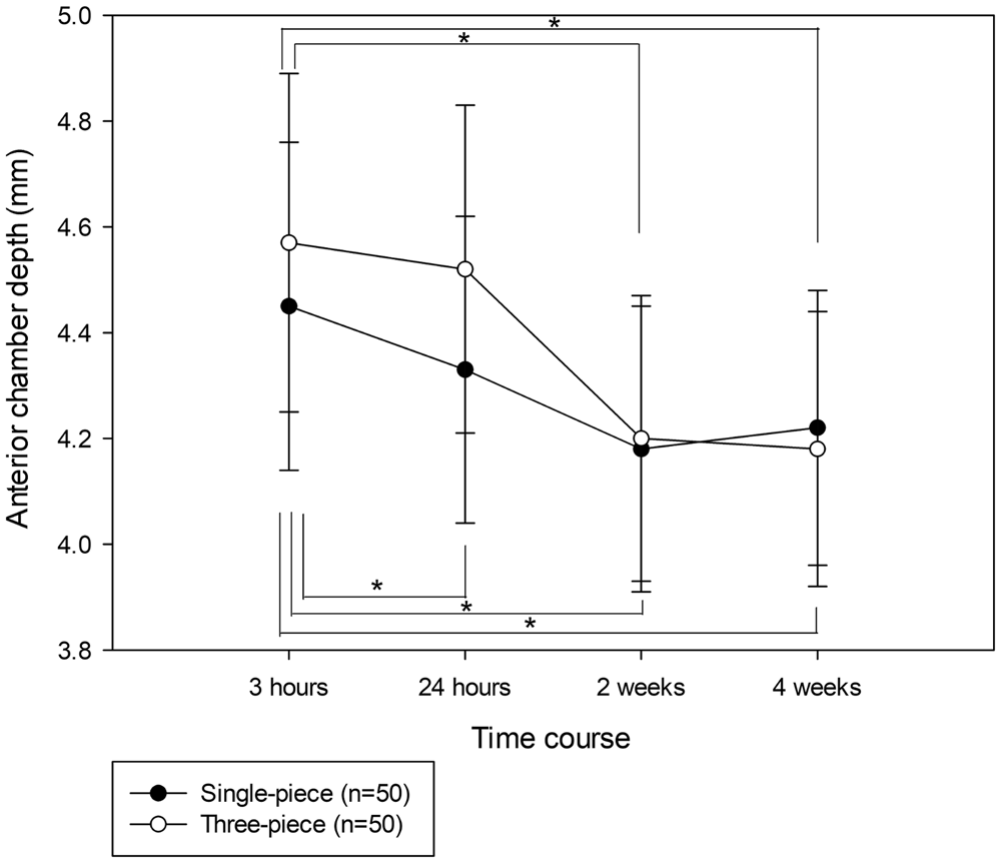
Anterior chamber depth (ACD) changes after single-piece and three-piece intraocular lens implantation. The abscissa represents perioperative time points and the ordinate represents ACD. Statistical analyses were performed using the Friedman repeated measures analysis of variance on ranks, followed by Dunnett’s method (*P < 0.05).

ACDs at 3 hours, 24 hours, 2 weeks, and 4 weeks after surgery were 4.45 ± 0.31 mm, 4.33 ± 0.29 mm, 4.18 ± 0.27 mm, and 4.22 ± 0.26 mm in eyes with the single-piece IOL, whereas they were 4.57 ± 0.32 mm, 4.52 ± 0.31 mm, 4.20 ± 0.27 mm, and 4.18 ± 0.26 mm in eyes with the three-piece IOL, respectively. ACDs of eyes with the single-piece IOL were significantly different (*P* = <0.001) among the four time points; further, ACD at 3 hours after surgery was significantly deeper (*P* < 0.05) than ACDs at the other three time points (Fig. 3). ACDs of eyes with the three-piece IOL were significantly different (*P* = <0.001) among the four time points; moreover, ACD at 3 hours after surgery was significantly deeper (*P* < 0.05) than ACDs at 2 and 4 weeks after surgery. ACD at 24 hours after surgery was significantly deeper (*P* = 0.009) in eyes with the three-piece IOL than in eyes with the single-piece IOL.

### Spherical Equivalent and Uncorrected Distance Visual Acuity

**Figure 4:**
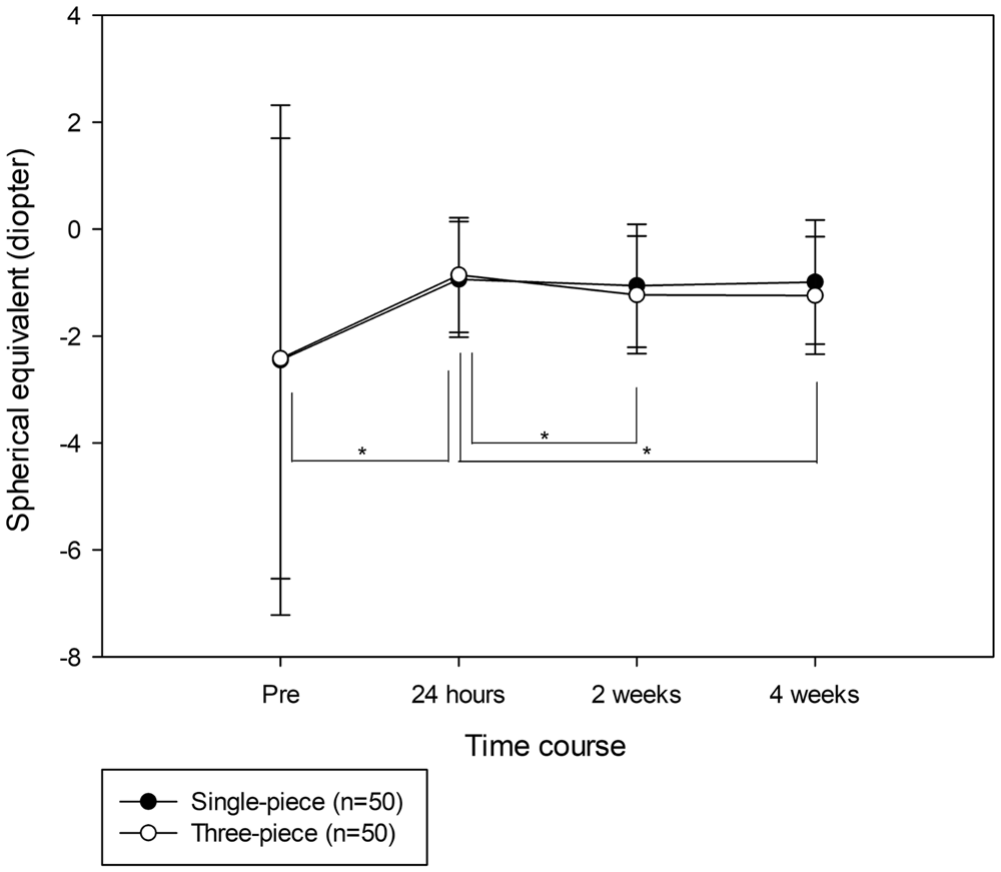
Spherical equivalent (SE) changes before and after single- and three-piece intraocular lens implantation. The abscissa represents perioperative time points and the ordinate represents the SE. Statistical analyses were performed using the Friedman repeated measures analysis of variance on ranks, followed by Dunnett’s method (*P < 0.05).

Pre-operative SEs, as well as those at 24 hours, 2 weeks, and 4 weeks after surgery were −2.45 ± 4.76 D, −0.94 ± 1.08 D, −1.06 ± 1.15 D, and −0.99 ± 1.16 D in eyes with the single-piece IOL, whereas they were –2.42 ± 4.12 D, −0.86 ± 1.07 D, −1.23 ± 1.10 D, and −1.24 ± 1.10 D in eyes with the three-piece IOL, respectively. SEs in eyes with the single-piece IOL were not significantly different among the four different time points. Conversely, there was a significant difference (*P* = <0.001) in the SEs of eyes with the three-piece IOL among the four time points, and the absolute value of the SE at 24 hours after surgery was significantly smaller (*P* < 0.05) than SEs at the other three time points (Fig. 4). Thus, eyes with the three-piece IOL demonstrated a statistically significant, approximately 0.4-D, myopic shift in SE. There were no significant differences in SE between eyes with single- and three-piece IOLs in any comparison of time points. Postoperative uncorrected distance visual acuities (UCDVAs) were significantly better (*P* < 0.05) than preoperative UCDVAs in both eyes with single- and three-piece IOLs. There were no significant differences in UCDVA between eyes with single- and three-piece IOLs in any comparison of time points.

### Angularity of IOL Loops and Coverage of Anterior IOL Surface by Capsulorhexis Edge

Angularity of the haptic-optic junction of IOL at postoperative 3 hours measured 97.0 ± 56.0 degrees in eyes with the single-piece IOL and 82.9 ± 52.8 degrees in eyes with the three-piece IOL. There was no significant difference in angularity between the two groups.

Complete coverage of anterior IOL surface by capsulorhexis edge was confirmed in 47 of 50 (94%) eyes with the single-piece IOL and in 49 of 50 (98%) eyes with the three-piece IOL. There was no significant difference in the ratio of anterior IOL surface coverage between the two groups.

## Discussion

In this study, we investigated short-term dynamics after single- and three-piece IOL implantation using SS-ASOCT, utilizing identical IOL material from a single manufacturer, after uneventful phacoemulsification. Repeatability of IOL decentration and tilt measurements, as well as of ACD measurements, by SS-ASOCT (CASIA2), were evaluated using the ICC. We found that intra- and interobserver reproducibility was almost perfect. These results suggest that SS-ASOCT may serve as a reliable tool for evaluation of IOL location.

Both single- and three-piece IOLs did not show any constant directionality of decentration (Fig. 1), whereas both IOLs exhibited a tendency to tilt toward the inferotemporal direction (Fig. 2). Kimura *et al*. investigated an IOL location at ≥1 week after surgery and found a similar tilt toward the inferotemporal direction^5^. Our time-course investigation revealed that both single- and three-piece IOLs had begun to tilt toward the inferotemporal direction as early as 24 hours after surgery. In fact, the azimuth of single-piece IOL tilt at ≥24 hours after surgery was significantly larger than the same azimuth measurement at 3 hours after surgery.

Absolute values of IOL decentration at 24 hours and 2 weeks after surgery were significantly larger in eyes with the single-piece IOL, compared with those with the three-piece IOL. This transient difference may be attributed to the softer haptic loops of the single-piece IOL, in comparison with those of the three-piece IOL; in contrast, there was no significant difference in the absolute value of decentration between the two IOLs at 4 weeks after surgery. There was no significant difference in IOL tilt values between eyes with single- and three-piece IOLs during the follow-up period. Longitudinal investigation with Scheimpflug videophotography revealed no significant differences in absolute values of IOL decentration or tilt between eyes with a single- or a three-piece IOL the broader postoperative period (3 days to 6 months)^1^. Taken together, these results suggest that measurements of IOL decentration and tilt in eyes with a single-piece IOL may be similar to measurements in eyes with a three-piece IOL, ≥4 weeks after surgery.

ACD at 24 hours after surgery was significantly deeper in eyes with the three-piece IOL, compared with ACD in eyes with the single-piece IOL; however, there was no significant difference in ACD between eyes with single- and three-piece IOLs at ≥2 weeks after surgery. Such drastic ACD changes in eyes with the three-piece IOL may arise from the characteristics of the haptic loops. There is a discrepancy in diameter between the IOL and the lens capsule. The overall diameter of the three-piece IOL was 13.0 mm, whereas lens capsule diameter has been reported as 10.0–11.0 mm^8,9^. Therefore, the loops are contracted by the lens capsule after surgery. The polymethyl methacrylate (PMMA) loops of the three-piece IOL are also more rigid than the acrylic loops of the single-piece IOL. In addition, the PMMA loops have an angulation of 10 degrees to maximize the contact area of the three-piece IOL with the posterior capsule after surgery, which is effective in preventing the development of posterior capsule opacity^10,11^. These characteristics of the haptic loops may make the three-piece IOL more easily affected by the discrepancy in diameter between the IOL and the lens capsule; in contrast, the more flexible acrylic loops of the single-piece IOL may absorb the contraction force of the empty capsular bag after surgery, resulting in reduced shifting, compared with the three-piece IOL. In fact, a previous study demonstrated more optic displacement along the optical axis when using three-piece IOLs, compared with single-piece IOLs^12^. As a result, shallowing of the ACD resulted in an approximately 0.4-D myopic shift of SE in eyes with the three-piece IOL.

Importantly, we found no significant differences in SE and UCDVA between eyes with single- and three-piece IOLs at any postoperative time point. These results suggest that, although both single- and three-piece IOL location varied transiently during the period immediately following surgery, IOL locations were similar at 4 weeks after surgery and did not exhibit any statistical difference in visual function.

There are some limitations in this study. This was a single-centre, prospective, randomized comparative study, with a short-term follow-up period. Thus, we did not evaluate IOL locations beyond 4 weeks postoperatively. In addition, this study excluded eyes with ocular pathologies other than cataract. Thus, further studies are needed to investigate IOL locations in eyes with cataract accompanied by pathology (e.g., pseudoexfoliation syndrome^13^, angle-closure glaucoma^14^, and diabetes^15^). Nevertheless, the data obtained from this relatively large number of patients who were arranged randomly provides new information about IOL locations after cataract surgery.

In conclusion, we compared postoperative dynamics of single- and three-piece IOLs after surgery, using the SS-ASOCT; this technology enables high reproducibility in detection of the IOL position. We found that, although both single- and three-piece IOL location varied transiently during the period immediately after surgery, IOL locations were similar at 4 weeks after surgery and did not exhibit any statistical difference in visual function.

## Methods

This was a prospective, randomized, comparative observational study. This study was registered in the University Hospital Medical Information Network on 12 January 2018 (registration number: 000030788). The institutional review board of Hayashi Eye Hospital approved this study, which adhered to the tenets of the Declaration of Helsinki. All patients provided written informed consent after receiving an explanation of the nature and possible consequences of the study. We can comply with the publication’s requirements for sharing materials.

### Patients

All patients who were scheduled for bilateral cataract surgery at Hayashi Eye Hospital between March 2016 and April 2017 were consecutively screened for inclusion in this study. Exclusion criteria consisted of ocular pathology other than cataract that might affect visual acuity; history of ocular surgery and/or inflammation; eyes scheduled for extracapsular or intracapsular cataract extraction; ≤6.0 mm pupil diameter after mydriasis; pseudoexfoliation syndrome; diabetes; and patients not available for follow-up. Screening continued until 50 patients were recruited prior to undergoing phacoemulsification and IOL implantation.

The day before surgery, patients were randomly assigned to one of two groups: 25 patients who were to receive a single-piece acrylic foldable spherical IOL (SN60AT; Alcon, Fort Worth, TX, USA) in the right eye and a three-piece acrylic foldable spherical IOL (MA60AC; Alcon) in the left eye; and 25 patients who were to receive the three-piece IOL in the right eye and the single-piece IOL in the left eye.

The SN60AT has an optic diameter of 6.0 mm and an overall diameter of 13.0 mm. The soft acrylic STABLEFORCE^TM^ modified-L loops have no angulation. Although the MA60AC has the same optic and overall diameters as the SN60AT, the PMMA-modified C-loops have an angulation of 10 degrees.

A clinical research coordinator generated randomization codes with equal numbers, using random number tables, and concealed the assignment schedule until all data were collected to ensure proper blinding during allocation. Patients and ocular examiners were not aware of the type of IOL implanted in each eye.

### Surgery

All surgeries were performed by one experienced surgeon (K.H.), who did not know the type of IOL before the day of surgery. Target postoperative refractive error ranged from −0.04 to −2.66 D, based on IOLMaster^®^ 700 (Carl Zeiss Meditec, Inc., Dublin, CA, USA) measurements, and did not significantly differ between eyes with single- and three-piece IOLs.

A 2.4-mm temporal corneal incision was made. A continuous curvilinear capsulorhexis, approximately 5.5 mm in diameter, was created to completely cover the anterior IOL surface with the capsulorhexis edge by using a 25-gauge bent sharp needle. After hydrodissection and delineation, phacoemulsification of the nucleus and aspiration of the residual cortex were performed. The lens capsule was inflated with sodium hyaluronate 1% (Hyaguard^®^; Nitten Pharmaceutical CO., Ltd., Nagoya, Japan), after which the IOL was placed randomly in the capsular bag by using an injector. After IOL implantation, the viscoelastic material was thoroughly evacuated. No sutures were placed in any eye. All surgeries were uneventful, and IOLs were implanted accurately within the capsular bag.

### Outcome Measures

SE and UCDVA were measured before surgery, as well as at 24 hours, 2 weeks, and 4 weeks after surgery. SE values were determined as the spherical power minus half of the cylindrical power (D). UCDVA was measured using a Landolt C acuity chart and converted to the logarithm of the minimum angle of resolution (logMAR) units for statistical analyses.

Measurements of IOL decentration and tilt, and measurements of ACD, were performed under well-dilated conditions at 3 hours, 24 hours, 2 weeks, and 4 weeks after surgery, using the CASIA2; this instrument performed a three-dimensional analysis with 16 different angles of AS-OCT images, then automatically measured IOL decentration and tilt relative to the visual axis and the ACD.

Degree of IOL decentration was presented as the absolute value (mm) relative to the visual axis, along with its azimuth (degree). Degree of IOL tilt was presented as the absolute value (degree) relative to the visual axis, along with its azimuth (degree). A coordinate system was utilized to show the azimuth angle in which the zero-degree direction indicates the observer’s right side and 90-degree direction indicates the superior direction.

CASIA2 images taken postoperatively were also used to investigate whether there was a significant difference in the angularity of the haptic-optic junction of IOL between eyes with single- and three-piece IOLs and whether there was a significant difference in the ratio of complete coverage of the anterior IOL surface by the capsulorhexis edge between the two groups.

### Intraobserver and Interobserver Reproducibility

Intraobserver and interobserver reproducibilities—for measurements using the SS-ASOCT—were evaluated based on the ICC. There are three types of ICCs (class 1 to 3), according to Shrout and Fleiss^16^; we chose the class 1 ICC for intraobserver reproducibility and class 2 ICC for interobserver reproducibility.

Three independent examiners measured the degree of IOL decentration and tilt, as well as the ACD, in 17 patients who had undergone uneventful phacoemulsification and IOL implantation at Hayashi Eye Hospital. Each patient underwent five independent sets of measurements by three examiners. The intraobserver correlation coefficient was calculated by comparing the five sets of measurements obtained by each examiner for the same eye, whereas the interobserver correlation coefficient was calculated by comparing the first value of each examiner’s five measurements of a given eye with the first value of the same eye obtained by the other two examiners.

### Statistical Analysis

Data are presented as the mean ± standard deviation.

The significance of differences between right and left eyes was determined by paired t-test if the data were normally distributed, and by Wilcoxon signed rank test if they were not. Significant differences in the ratios between eyes with single- and three-piece IOLs were determined with Fisher’s exact test. Friedman repeated measures analysis of variance on ranks was performed to compare clinical conditions within the same subjects at different perioperative periods because the data were not normally or equally distributed. This was followed by Dunnett’s method to detect significant differences between each time point and immediate postoperative values. These statistical analyses were performed using SigmaPlot version 12.0 for Windows (Systat Software, Inc., San Jose, CA, USA). ICC was analysed using Stata version 15 (STATA Corp., College Station, TX, USA).

A *P*-value <0.05 was considered to be statistically significant.

### Data availability

The datasets generated during and/or analysed during the current study are available from the corresponding author on reasonable request.
